# Simvastatin induced ferroptosis for triple-negative breast cancer therapy

**DOI:** 10.1186/s12951-021-01058-1

**Published:** 2021-10-09

**Authors:** Xianxian Yao, Ruihong Xie, Yongbin Cao, Jing Tang, Yongzhi Men, Haibao Peng, Wuli Yang

**Affiliations:** 1 State Key Laboratory of Molecular Engineering of Polymers & Department of Macromolecular Science, Fudan University, Shanghai, 200433 China; 2 Department of Materials Science and Engineering, Stanford University, Stanford, CA USA; 3 Shanghai General Hospital, Shanghai Jiao Tong University School of Medicine, Shanghai, 200080 China; 4 Institute for Translational Brain Research, Fudan University, Shanghai, 200032 China

**Keywords:** Triple-negative breast cancer, Ferroptosis, Simvastatin, Long circulation, Controlled release

## Abstract

Triple-negative breast cancer (TNBC), a management of aggressive breast cancer, remains an unmet medical challenge. Although a wave of efforts had spurred to design novel therapeutic method of TNBC, unpredictable prognosis with lacking effective therapeutic targets along with the resistance to apoptosis seriously limited survival benefits. Ferroptosis is a non-apoptotic form of cell death that is induced by excessive lipid peroxidation, which provide an innovative way to combat cancer. Emerging evidence suggests that ferroptosis plays an important role in the treatment of TNBC cells. Herein, a novel ferroptosis nanomedicine was prepared by loading simvastatin (SIM), a ferroptosis drug, into zwitterionic polymer coated magnetic nanoparticles (Fe_3_O_4_@PCBMA) to improve the therapeutic effect of TNBC. The as-obtained Fe_3_O_4_@PCBMA-SIM nanoparticles demonstrated more cytotoxicity against MDA-MB-231 than MCF-7 due to the higher expression of 3-hydroxy-3-methyl-glutaryl-coenzyme A reductase (HMGCR), which demonstrated that statins could effectively kill TNBC. Further experiments showed that SIM could inhibit the expression of HMGCR to downregulate the mevalonate (MVA) pathway and glutathione peroxidase 4 (GPX4), thereby inducing cancer cell ferroptosis. What’s more, PCBMA endows Fe_3_O_4_@PCBMA longer blood circulation performance to enhance their accumulation at tumor sites. Given that Fe_3_O_4_ have proven for clinical applications by the U.S. Food and Drug Administration (FDA) and SIM could induce cancer cell ferroptosis, the developed Fe_3_O_4_@PCBMA-SIM nanosystem would have great potential in clinics for overcoming the drug resistance brought about by apoptotic drugs to cancer cells.

**Supplementary Information:**

The online version contains supplementary material available at 10.1186/s12951-021-01058-1.

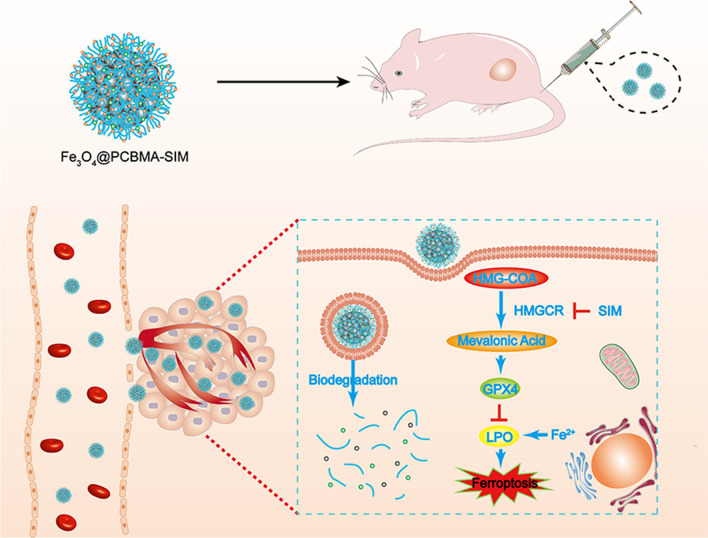

## Introduction

Breast cancer has the highest fatality rate in women worldwide [1]. Among all types and forms of breast cancer, triple negative breast cancer (TNBC) is the most aggressive and heterogeneous subtype due to lack of estrogen and progesterone receptor expressions, which has been an unmet medical challenge in clinic [2, 3]. The distinct cellular phenotype with lack of receptor or target makes chemotherapy as an excellent treatment for TNBC. Nonetheless, the drug resistance and toxic side effects resulting from anti-cancer drugs lead to the failure of cancer chemotherapy, which due to the acquired or intrinsic resistance of cancer cells to apoptosis [4]. Hence, develop effective non-apoptotic treatment strategies has become an urgent need for the treatment of TNBC.

Ferroptosis is a form of iron-dependent cell death induced by excessive lipid peroxidation that distinct from the traditional apoptosis and necrosis [5]. Since the term ferroptosis was proposed in 2012, the unique mechanism of ferroptosis has attracted increasing attention in the field of antitumor therapy [6]. The redox-active iron (Fe^2+^) is the key elements of ferroptosis and this process characterized by direct or indirect inhibition of glutathione peroxidase 4 (GPX4), lipid repair enzyme, and lipid hydroperoxides (LPO) accumulation [7, 8]. Consequently, the intracellular accumulation of LPO leading to impaired cell structure and integrity [9]. The inactivation of GPX4 was produced in the presence of erastin analogs or the direct GPX4 inhibitor [10], such as RSL-3 [11], sorafenib [9], statins [12] and so on. Erastin is a low molecular chemotherapeutics agent and TNBC cells have been reported to be sensitive to erastin‐induced ferroptosis using xCT cystine/glutamate antiporter as a common therapeutic target for TNBC [13, 14]. To date, several mechanisms leading to iron and reactive oxygen species (ROS) metabolism of ferroptosis have been addressed, but the mechanisms of ferroptosis in breast cancer cells especially in TNBC has hardly been reported [15]. Moreover, it is still challenging to engineer the iron ion delivery system to enhancement the effect of ferroptosis. Therefore, improving the efficiency of iron ion delivery in the process of ferroptosis is of great significance to TNBC patient for cancer treatment and drug design [10].

Statins are a class of low molecular weight drugs that have been approved for clinical control of hypercholesterolemia [16]. Recent study has shown that statins have a potential role in cancer prevention due to their ability to inhibit proliferation, angiogenesis and inflammation [12, 17]. Joseph et al*.* reported that statins could lower cholesterol through inhibit 3-hydroxy-3-methyl-glutaryl-coenzyme A reductase (HMGCR) to regulate the mevalonate (MVA) pathway [18]. Moreover, it was reported that isopentenyl pyrophosphate participant in the biosynthesis of GPX4 through MVA pathway and HMGCR played a vital role in the synthesis of isopentenyl pyrophosphate [19, 20]. So far, ferroptosis has been thought to induce cancer cell oxidative damage by controlling the phospholipid hydroperoxide-reducing enzyme GPX4 [19, 21, 22]. Therefore, it will be of great significance if statins could kill TNBC through the way of ferroptosis. However, statins as a kind of small molecule drug is metabolized quickly and few drugs accumulate to the lesion site, severely reducing the effectiveness of treatment. Nanoscale sized materials as an excellent carrier could deliver antitumor drugs to tumor tissues through passively target [23]. But most of nanoparticles rapidly cleared by the reticuloendothelial system as exogenous invaders that affect the percentage of administered nanoparticles reaching in tumor site and limited therapeutic effect [24, 25]. Therefore, development of novel nanomedicine with long circulation that can enhance the statins accumulation for in vivo TNBC therapy is urgently required.

In this work, we present the construction of zwitterionic polymer coating of ferroferric oxide nanoparticles (Fe_3_O_4_@PCBMA) to prolong its blood circulation. Simvastatin (SIM), a ferroptosis drug, could be loaded into Fe_3_O_4_@PCBMA (Fe_3_O_4_@PCBMA-SIM). MDA-MB-231, a TNBC model, and MCF-7, a normal breast cancer cell model, were used to evaluate the cancer cell killing efficiency. The results showed SIM have more cytotoxicity against MDA-MB-231 than MCF-7, which demonstrated that statins could effectively kill TNBC. In addition, the western blot result illustrated SIM could through inhibit HMGCR to modulate the MVA pathway and deactivate GPX4. With the inherited blood circulation property and ferroptosis effect, the in vivo therapeutic efficiency of Fe_3_O_4_@PCBMA-SIM was evaluated through building MDA-MB-231 tumor-bearing mice. Our finding highlights that Fe_3_O_4_@PCBMA-SIM exhibit an excellent tumor suppression, which will open an avenue of TNBC therapy.

## Results and discussion

### The preparation of Fe_3_O_4_@PCBMA

**Scheme 1:**
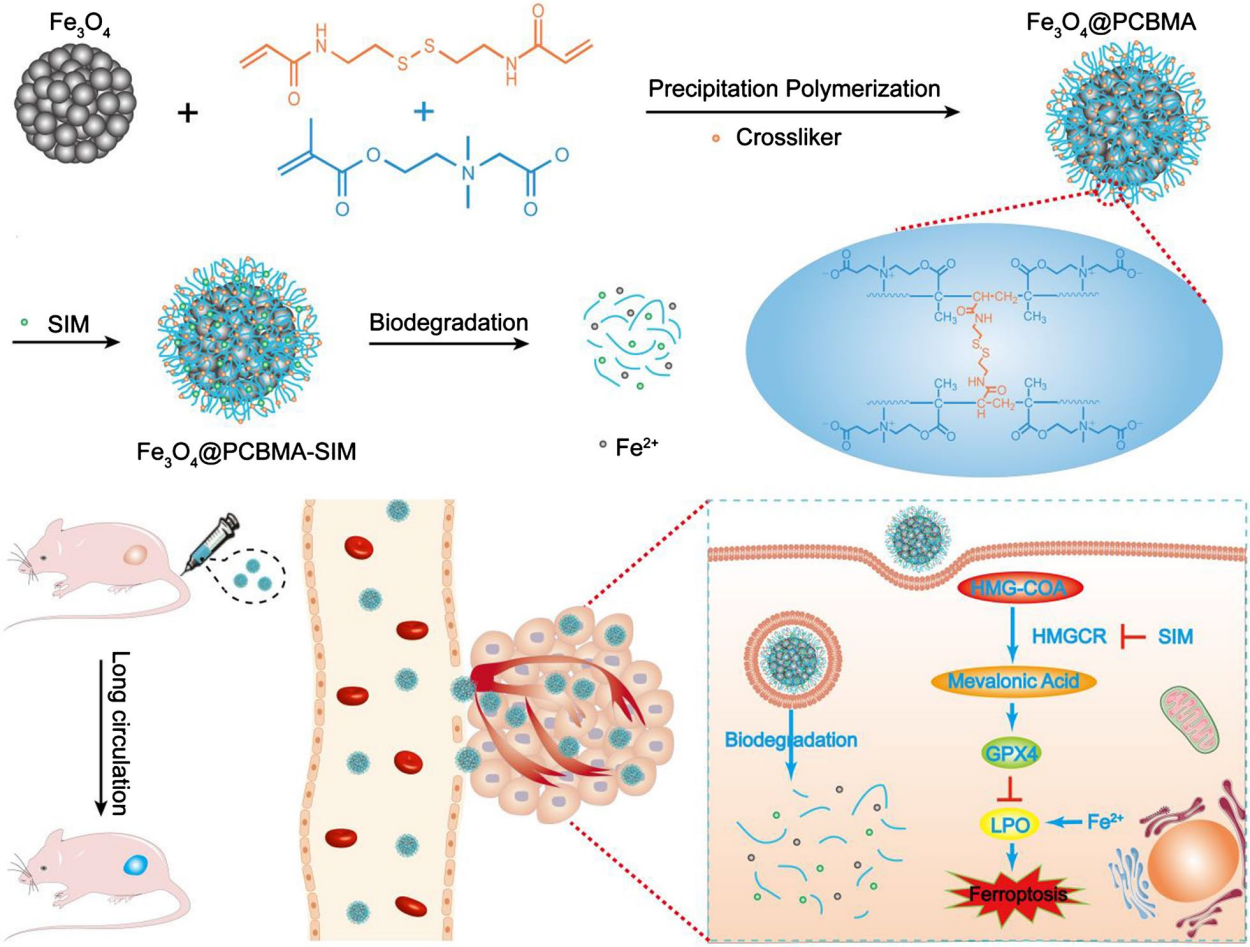
Schematic illustration of multifunctional nanoplatform for ferroptosis

**Fig. 1:**
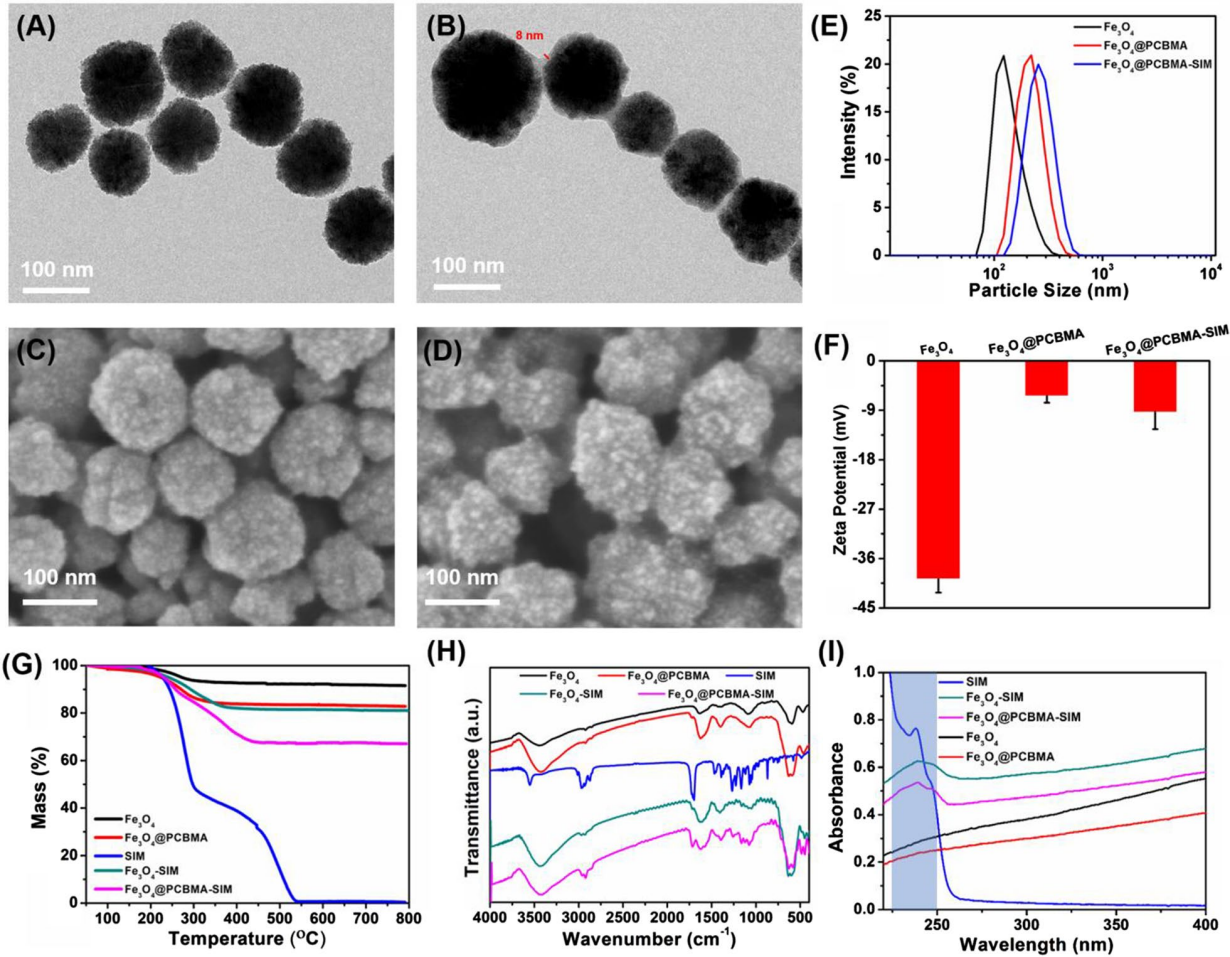
TEM images of Fe_3_O_4_ (**A**) and Fe_3_O_4_@PCBMA (**B**) nanoparticles; SEM images of Fe_3_O_4_ (**C**) and Fe_3_O_4_@PCBMA nanoparticles (**D**); DLS diameters of Fe_3_O_4_, Fe_3_O_4_@PCBMA, and Fe_3_O_4_@PCBMA-SIM nanoparticles (**E**); Zeta potential of Fe_3_O_4_, Fe_3_O_4_@PCBMA, and Fe_3_O_4_@PCBMA-SIM nanoparticles (**F**); TGA analysis of Fe_3_O_4_, Fe_3_O_4_@PCBMA, SIM, Fe_3_O_4_-SIM and Fe_3_O_4_@PCBMA-SIM nanoparticles measured in air (**G**); FT-IR spectra of Fe_3_O_4_, Fe_3_O_4_@PCBMA, SIM, Fe_3_O_4_-SIM and Fe_3_O_4_@PCBMA-SIM nanoparticles (**H**); UV–vis spectra of SIM, Fe_3_O_4_-SIM, Fe_3_O_4_@PCBMA-SIM, Fe_3_O_4_ and Fe_3_O_4_@PCBMA nanoparticles (**I**)

Carboxybetaine methacrylate (CBMA) was synthesized according to the reported method [26]. Then, we encapsulated Fe_3_O_4_ with poly (carboxybetaine methacrylate) (PCBMA) for enabling longer blood circulation performance [27]. The preparation process of Fe_3_O_4_@PCBMA was illustrated in Scheme 1. Before encapsulation, carbon–carbon double bond was firstly modified to magnetic nanoparticle, which was proved at the strong peaks centered of 1717 cm^−1^ by fourier transform infrared spectroscopy (FTIR) (Additional file 1: Fig. S1). Then, 3-aminopropyltriethoxysilane-modified Fe_3_O_4_ (Fe_3_O_4_-MPS) nanoparticles were coated with PCBMA network by reflux precipitation polymerization method [28]. Transmission electron microscope (TEM) images of Fe_3_O_4_ and Fe_3_O_4_@PCBMA with uniformed size displayed spherical morphology and the obvious polymer layer (Fig. 1A, B). In addition, the smooth surface of nanoparticles after coating from the scanning electron microscope also indicated the form of core–shell structure (Fig. 1C, D). It could be seen from the TEM image that the thickness of the zwitterionic polymer layer was about 8 nm. Meanwhile, the hydrate particle size of Fe_3_O_4_@PCBMA was a little bigger than Fe_3_O_4_ measured by dynamic light scattering (DLS), which was further prove the successful coating (Fig. 1E). In addition, there was no different of DLS particle size that Fe_3_O_4_@PCBMA dispersed in aqueous, phosphate buffer solution (PBS), bull serum albumin (BSA) and culture medium Dulbecco Minimum Essential Medium (DMEM), which indicated Fe_3_O_4_@PCBMA have the good stability (Additional file 1: Fig. S3). Furthermore, the zeta potential of Fe_3_O_4_@PCBMA increased to zero after coating, which due to the equal positive and negative of PCBMA (Fig. 1F). Therefore, the above results indicated the successful fabrication of Fe_3_O_4_@PCBMA nanoparticles. And then the SIM loading property was studied. After SIM loading, the morphology and size of the nanoparticles barely changed, indicating the stable of the drug-loaded nanoparticles (Additional file 1: Fig. S2). The FTIR spectra of the Fe_3_O_4_-SIM and Fe_3_O_4_@PCBM-SIM appeared new bands in the 1300–1000 cm^−1^ region corresponding to C–O–C of SIM. In addition, there are 11.4% weight loss of PCBMA at 200–400 °C and 15.7% weight loss of SIM at 200–350 °C showed by thermogravimetric analysis (TGA) of Fe_3_O_4_@PCBMA-SIM. Moreover, Fig. 1I showed the UV–vis absorbance spectra of the nanoparticles before and after loading SIM. The absorbance peak of Fe_3_O_4_-SIM and Fe_3_O_4_@PCBMA-SIM at 238 nm were attributed to the SIM characteristic absorbance, which indicated the SIM successful loading. These results suggested the successful fabrication of Fe_3_O_4_@PCBM-SIM through PCBMA coating and SIM loading.

### The biodegradation and controlled drug release

**Fig. 2:**
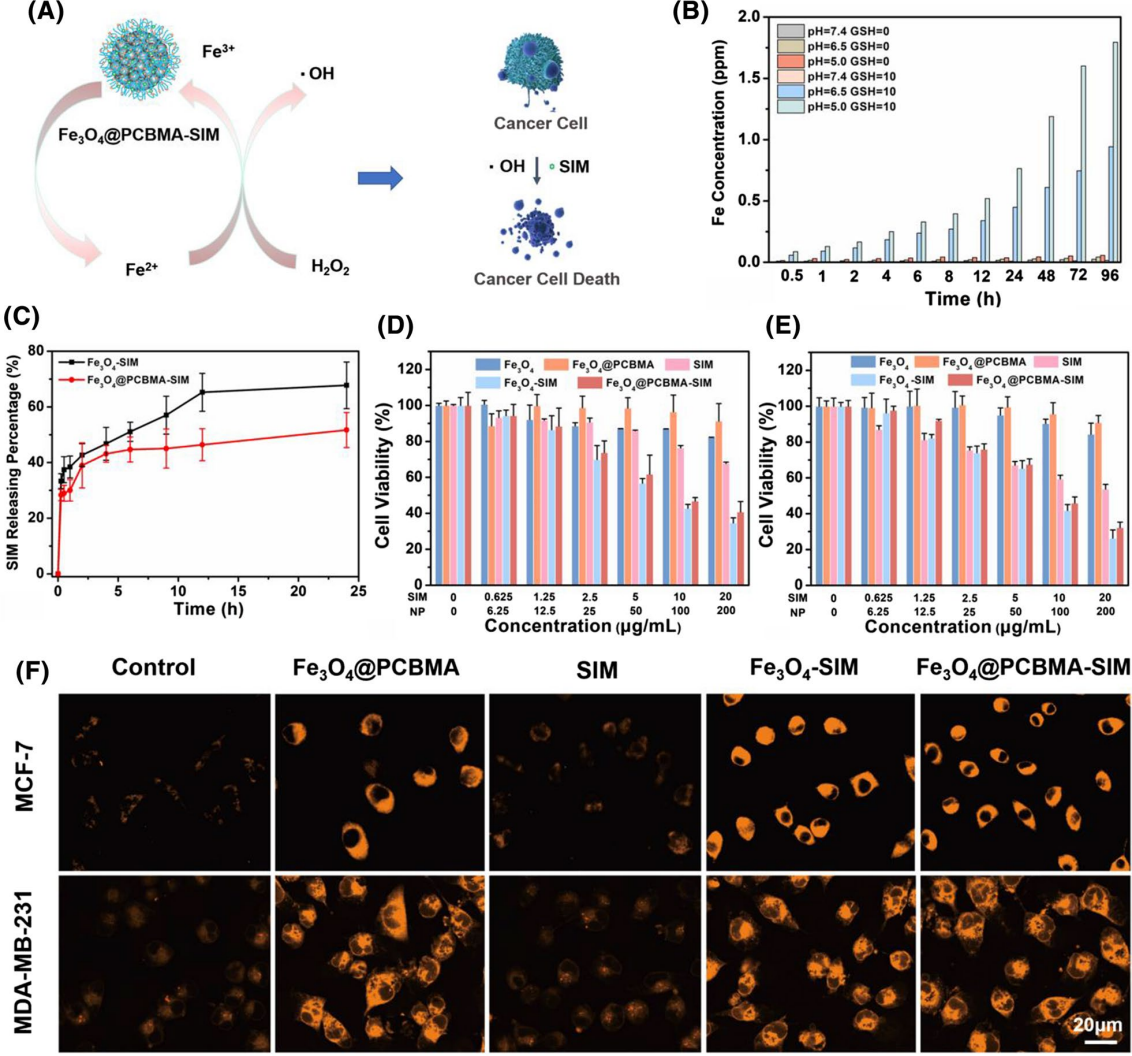
Schematic illustration of Fe_3_O_4_@PCBMA-SIM nanoparticles killing cancer cells (**A**); The degradation property of Fe_3_O_4_@PCBMA nanoparticles dispersed in different concentrations of GSH (0 mM and 10 mM) and pH values (5.0, 6.5 and 7.4) (**B**); Cumulative SIM releasing from Fe_3_O_4_-SIM and Fe_3_O_4_@PCBMA-SIM nanoparticles dispersed in 10 mM GSH (**C**); Cell viability of the MCF-7 (**D**) and MDA-MB-231 (**E**) cells after 48 h incubation with Fe_3_O_4_, Fe_3_O_4_@PCBMA, SIM, Fe_3_O_4_-SIM and Fe_3_O_4_@PCBMA-SIM suspensions; CLSM of control, Fe_3_O_4_@PCBMA, free SIM, Fe_3_O_4_-SIM and Fe_3_O_4_@PCBMA-SIM nanoparticles after uptake by MCF-7 and MDA-MB-231 cells (**F**)

A sustainable and efficient fenton reaction nanoplatform for tumor therapy was developed based on Fe_3_O_4_ nanoparticle. As described in Fig. 2A, Fe_3_O_4_@PCBMA-SIM nanoparticles could trigger more ROS generation in response to tumor microenvironment and release large amounts of Fe^2+^ for further promoting cancer cell death. In addition, the concentration of glutathione (GSH) in cancer cell was ranging from 2 to 10 mM and the pH was 6.5–5.0 in lysosomes of cancer cell and 7.4 in normal tissues [29, 30]. Therefore, the biodegradability of Fe_3_O_4_ and Fe_3_O_4_@PCBMA were investigated. Certified by the inductively coupled plasma spectrometry (ICP-AES), the degradation property of Fe_3_O_4_@PCBMA were increased by the increased concentrations of GSH and as the pH value of PBS decreased. After 96 h, the Fe concentration was only 1.8 µg/mL in the pH 5.0 buffer solution with GSH of 10 mM and there was only few Fe in neutral environment with GSH of 10 mM, implying that Fe_3_O_4_@PCBMA could be decomposed into iron ions (Fig. 2B). The degradation property of Fe_3_O_4_ was similar to Fe_3_O_4_@PCBMA and the Fe concentration (2.3 µg/mL) in the pH 5.0 buffer solution with GSH of 10 mM was higher than Fe_3_O_4_@PCBMA, which due to the existence of PCBMA in the shell (Additional file 1: Fig. S4). Therefore, SIM would be released faster after phagocytosis by cancer cells in theory.

To evaluate drug release behavior, we first measured the drug loading ratios of SIM by UV–vis absorbance spectra at 238 nm. The UV–vis calculated that SIM was loaded in Fe_3_O_4_ and Fe_3_O_4_@PCBMA with contents of 10 and 15% respectively. Then the mass ratio of nanoparticles to SIM was deliberately controlled to 10% for the consistency of subsequent experiments. Afterwards, the drug release property of Fe_3_O_4_-SIM and Fe_3_O_4_@PCBMA-SIM nanoparticles were studied by dispersing the nanoparticles into glutathione (GSH, 10 mM). As shown in Fig. 2C, the SIM releasing amount increased with incubating time and there were about 70% SIM released from Fe_3_O_4_-SIM and 55% SIM released from Fe_3_O_4_@PCBMA-SIM over 24 h, indicating a distinct rapid release behavior. Therefore, Fe_3_O_4_-SIM and Fe_3_O_4_@PCBMA-SIM could release SIM under the microenvironments of cancer cell.

### In-vitro cytotoxicity and cell uptake of Fe_3_O_4_@PCBMA-SIM

It is well known that TNBC is very difficult to treatment owing to its heterogeneity, molecular variability, and stemness [31]. It is very significant to develop sufficient drug to kill TNBC. The viability of breast cancer cell (MCF-7) and triple-negative breast cancer cell (MDA-MB-231) treated with Fe_3_O_4_@PCBMA-SIM were investigated in this study adopting the cell counting kit-8 (CCK-8) assay. Before in vitro cytotoxicity experience, we first studied the biocompatibility. As shown in Fig. 2D, E, the viability of two cancer cells cultured in the presence of Fe_3_O_4_ were indicated slightly toxicity. After encapsulate PCBMA, there were negligible cytotoxicity of Fe_3_O_4_@PCBMA even though the concentration of nanoparticles were 200 µg/mL. In addition, free SIM, Fe_3_O_4_-SIM and Fe_3_O_4_@PCBMA-SIM displayed a concentration-dependent cytotoxicity against both MCF-7 and MDA-MB-231 cells, and free SIM, Fe_3_O_4_-SIM and Fe_3_O_4_@PCBMA-SIM were more cytotoxic to MDA-MB-231 cells than to MCF-7 cells, which proved that Fe_3_O_4_@PCBMA-SIM could effectively kill TNBC. For example, the cell viability of MDA-MB-231 was 54% after treated with free SIM (20 µg/mL), which was lower than that MCF-7 cells (68%). The cytotoxicity to MDA-MB-231 (32%) still higher than to MCF-7 cells (41%) after loaded to Fe_3_O_4_, probably due to the higher contribution of SIM and Fe_3_O_4_ to the system than free SIM. However, Fe_3_O_4_-SIM exhibited slightly higher cytotoxicity to two cancer cells than Fe_3_O_4_@PCBMA-SIM, which was due to the shell protection of PCBMA. In addition, the cell viability of MCF-7 cells and MDA-MB-231 cells with ferrous sulfate were measured. The cell viability was no obvious difference after ferrous sulfate added compared to control group whether ferrous sulfate added to MCF-7 cells or MDA-MB-231 cells, which confirmed that the iron ion has no cytotoxicity against MDA-MB-231 (Additional file 1: Fig. S5) and MCF-7 (Additional file 1: Fig. S6). Therefore, it can be concluded that SIM was higher sensitive to MDA-MB-231 and Fe_3_O_4_@PCBMA-SIM was an excellent strategy for the treatment of TNBC.

Further, the phagocytosis property of nanoparticles was investigated using FerroOrange as iron ion detection reagent in cells [32]. As shown in Fig. 2F, the orange fluorescence were obvious when MCF-7 and MDA-MB-231 cells treated with Fe_3_O_4_, Fe_3_O_4_-SIM and Fe_3_O_4_@PCBMA-SIM nanoparticles, whereas few orange fluorescence in control group and free SIM group, indicating that enormous amount of Fe^2+^ ions generated via nanoparticles. Therefore, Fe_3_O_4_@PCBMA-SIM nanoparticles could be degraded into iron ion after phagocytosed by cancer cells.

### In-vitro reactive oxygen species (ROS) generation

**Fig. 3:**
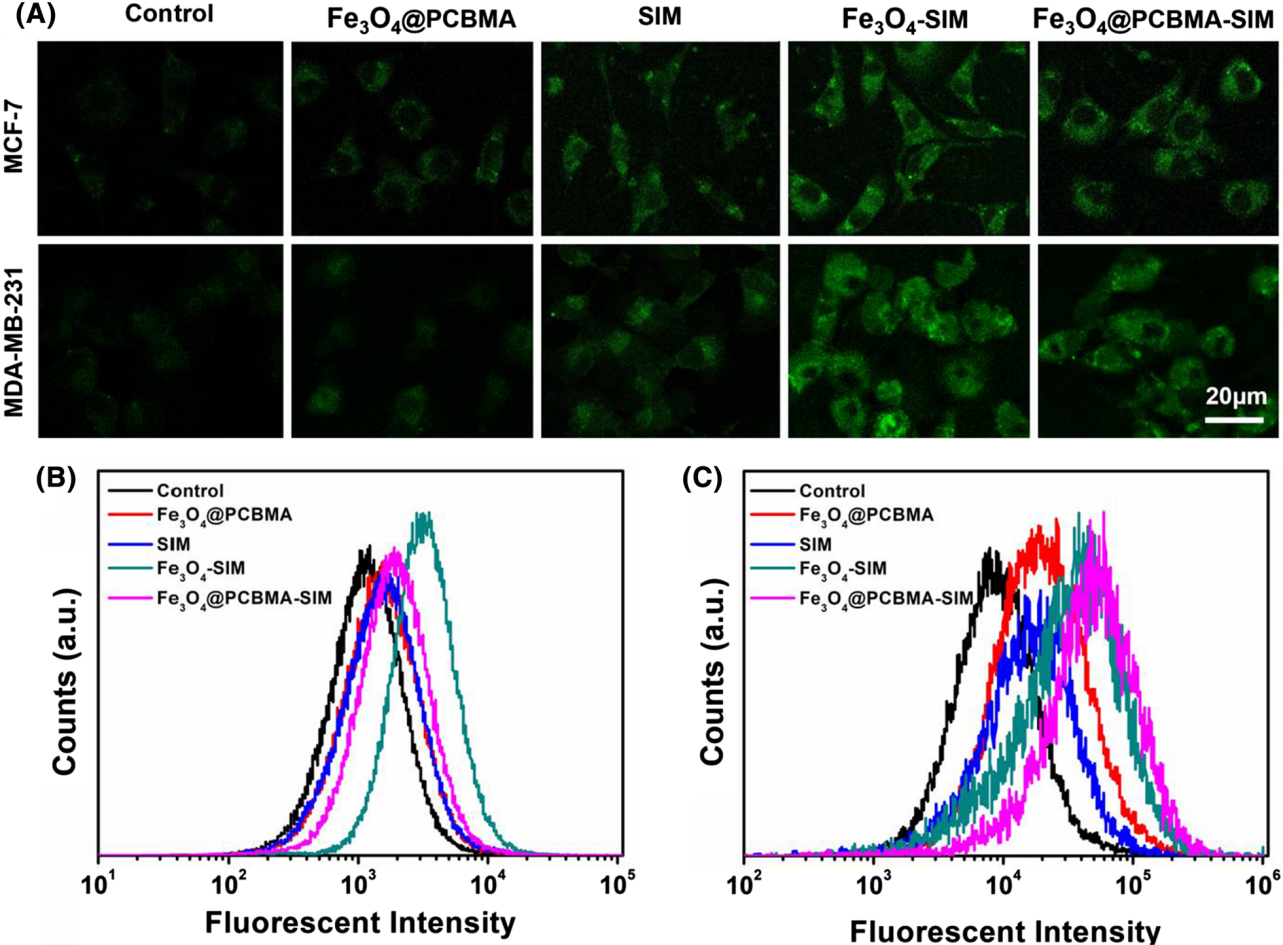
CLSM (**A**) and flow cytometry analysis (**B**, **C**) of reactive oxygen species (ROS) generation in MCF-7 and MDA-MB-231 cells

It was well-known that ROS could generated by ferrous ions via fenton reaction. To visually observe the generation of ROS, dichlorofluorescein diacetate (DCFH-DA) was used as a fluorescent probe to detect the generation of ROS [33]. As shown in Fig. 3A, MCF-7 and MDA-MB-231 cells showed weak fluorescence intensity incubated with Fe_3_O_4_@PCBMA due to the insufficient concentration of H_2_O_2_ to produce a small amount of ROS. The fluorescence slightly increased after incubation with free SIM, on account of the production of SIM through the MVA pathway. After treatment with Fe_3_O_4_-SIM and Fe_3_O_4_@PCBMA-SIM, the intensity of green fluorescence of cells was significantly enhanced. In addition, Fe_3_O_4_-SIM group and Fe_3_O_4_@PCBMA-SIM group exhibit stronger fluorescence intensity than free SIM group from the analysis of flow cytometry (Fig. 3B, C) both in MCF-7 cells and MDA-MB-231 cells, which indicated the enhanced production of ROS in cancer cells induced by Fe_3_O_4_-SIM or Fe_3_O_4_@PCBMA-SIM.

### In-vitro mechanism of ferroptosis

**Fig. 4:**
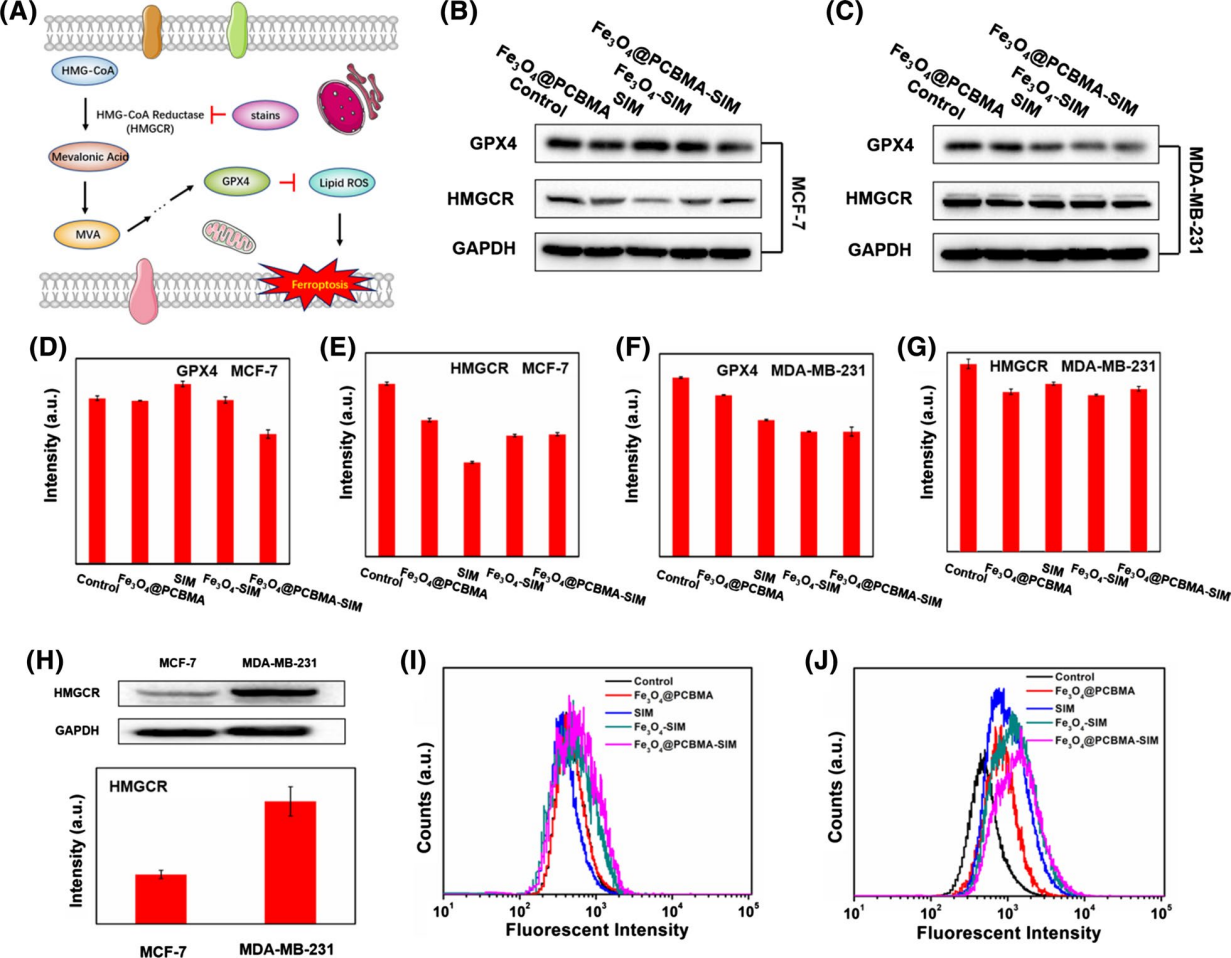
Schematic illustration of ferroptosis mechanism (**A**); The GPX4 and HMGCR protein expression of MCF-7 cells after treated with Fe_3_O_4_@PCBMA, SIM, Fe_3_O_4_-SIM and Fe_3_O_4_@PCBMA-SIM (**B**, **D**, **E**); The GPX4 and HMGCR protein expression of MDA-MB-231 cells after treatment with Fe_3_O_4_@PCBMA, SIM, Fe_3_O_4_-SIM and Fe_3_O_4_@PCBMA-SIM (**C**, **F**, **G**); The HMGCR protein expression of MCF-7 cells and MDA-MB-231 cells and the corresponding intensity (**H**); Flow cytometry analysis of lipid hydroperoxides (LPO) generation in MCF-7 (**I**) and MDA-MB-231 cells (**J**)

Statins, a small molecule potent inhibitors of the 3-hydroxy-3-methylglutaryl-coenzyme A reductase (HMGCR), could affect the MVA pathway to control the biosynthesis of cholesterol [34]. This process in suppression of some metabolites and inactivation of GPX4 [12, 35]. For further study the mechanism of ferroptosis induced by Fe_3_O_4_@PCBMA-SIM, western blot was used to study the MVA pathway. As shown in Fig. 4B, C, the expression of GPX4 and HMGCR protein in MCF-7 cells and MDA-MB-231 cells were influenced by the addition of SIM and Fe_3_O_4_. For HMGCR protein, both Fe_3_O_4_@PCBMA and SIM groups all decreased its expression compared with the control group and this lowering effect was obviously in MCF-7 cells (Fig. 4E, G). However, the expression of GPX4 in MCF-7 cells after treated with Fe_3_O_4_@PCBMA, SIM and Fe_3_O_4_-SIM were almost no difference and the expression of GPX4 protein decreased obviously after treated with Fe_3_O_4_@PCBMA and SIM, and further decreased after the addition of Fe_3_O_4_-SIM and Fe_3_O_4_@PCBMA-SIM in MDA-MB-231 cells, which was due to the synergy effect of SIM and Fe_3_O_4_ (Fig. 4D, F). In addition, we have also measurement the amount of HMGCR protein in two cancer cells to study the reason of this phenomenon. As shown in Fig. 4H, the HMGCR protein expression in MDA-MB-231 cells was much higher than in MCF-7 cells, which accounted for the unconspicuous inhibition effect of HMGCR protein. Therefore, the above results show that ferroptosis could occur through MVA pathway to inactivation of GPX4 in TNBC.

Moreover, the inactivation of GPX4 would inhibit the conversion of lipid peroxides into lipid alcohols and the lipid hydroperoxides (LPO) can be used as an important indicator of the ferroptosis [36]. Therefore, the inactivation of GPX4 could promote the accumulation of lipid peroxide level. As shown in Fig. 4I, J, the fluorescence intensity of cells in Fe_3_O_4_@PCBMA, SIM and Fe_3_O_4_@PCBMA-SIM groups showed stronger than that of the control group, which indicated more LPO production after nanodrugs effect. Unlike ROS, the basic LPO in MDA-MB-231 cells was higher than in MCF-7 cells and MDA-MB-231 cells produce more LPO than MCF-7 cells after incubation, which could account for the more cytotoxic of Fe_3_O_4_@PCBMA-SIM to MDA-MB-231 cells than to MCF-7 cells. Therefore, we could conclude that this nanoplatform could inhibit the expression of HMGCR to downregulate the mevalonate (MVA) pathway and glutathione peroxidase 4 (GPX4), thereby producing more LPO to induce cancer cell ferroptosis, as schematically illustrated in Fig. 4A.

### Pharmacokinetics and biodistribution

**Fig. 5:**
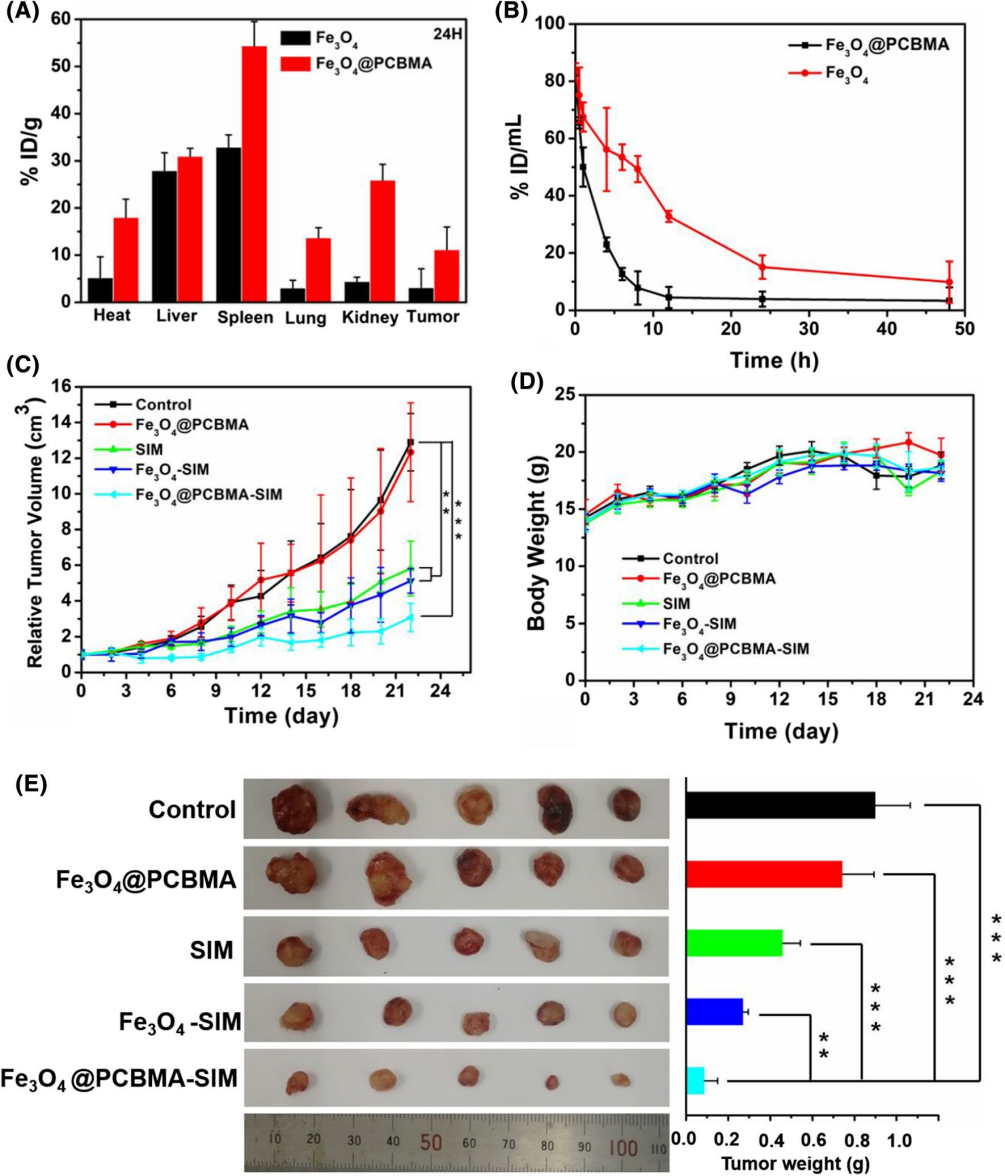
Biodistribution of Fe_3_O_4_ and Fe_3_O_4_ @PCBMA nanoparticles after 24 h injection (**A**); In vivo blood retention profiles of Fe_3_O_4_ and Fe_3_O_4_@PCBMA nanoparticles (1 mg/mL, 100 μL) (**B**); Tumor growth curves and body weight changes of nanomedicine. Treatments were performed on day 0, 3, 6 and 9. Error bars indicate the SD (n = 5) (**C**, **D**); Photographs (left) and weights (right) of tumors after therapy (**E**) *P < 0.05 **P < 0.01 ***P < 0.001

Nanoparticles could achieve better therapeutic efficacy compared with free drugs due to the high permeability and retention effect of solid tumors [37]. Nevertheless, the unsatisfactory tumor accumulation of nanoparticles is due to the undesirable blood circulation [31]. Therefore, design nanomedicine with prolonged blood circulation property is very important. In this study, we fabricated zwitterionic polymer coating of magnetic nanoparticles for enhancing their blood retention and effectively improved the therapeutic effect of TNBC. MDA-MB-231 tumor-bearing mice were used to study the accumulation property of Fe_3_O_4_@PCBMA in vivo. After 12, 24 and 48 h injection, visceral organs and tumors were taken out. Then, Fe content were measured using ICP-AES. The accumulation of Fe_3_O_4_@PCBMA and Fe_3_O_4_ in tumors at 12 h post injection were 12.6 ± 2.1% ID/g and 5.2 ± 1.2% ID/g respectively (Additional file 1: Fig. S7). As shown in Fig. 5A, the accumulation of Fe_3_O_4_@PCBMA just decreased to 11.1 ± 4.8% ID/g at the tumor site after 24 h injection and the accumulation of Fe_3_O_4_ reduced to 3.1 ± 3.9% ID/g, which indicated that there are more Fe_3_O_4_@PCBMA intratumor accumulation than Fe_3_O_4_. Interestingly, the accumulation of Fe_3_O_4_@PCBMA was still 8.1 ± 0.7% ID/g 4 at 48 h post injection (Additional file 1: Fig. S8). Moreover, in addition to the decreased nanoparticles at the tumor site, the residual amounts of nanoparticles in liver and lung also significantly reduced, while the uptake increased in kidney within 24 h, indicating the better metabolic performance of Fe_3_O_4_@PCBMA.

For further study the nanoparticle retention in blood, pharmacokinetics studies were performed for Fe content measurement using ICP-AES. As shown in Fig. 5B, Fe_3_O_4_ in blood was decreased to 3.9 ± 2.6% ID/mL after 24 h intravenous injection. In contrast, about 15.1 ± 4.1% ID/mL of Fe_3_O_4_@PCBMA were still in blood circulation. Moreover, there was 9.9 ± 7.1% ID/mL of Fe_3_O_4_@PCBMA nanoparticles stayed in blood after 48 h injection. A significant increase indicated that zwitterionic polymer could prolong blood circulation time of nanoparticles in vivo and it also proved the successfully synthesis of Fe_3_O_4_@PCBMA.

### In vivo antitumor combined therapy efficacy

**Fig. 6:**
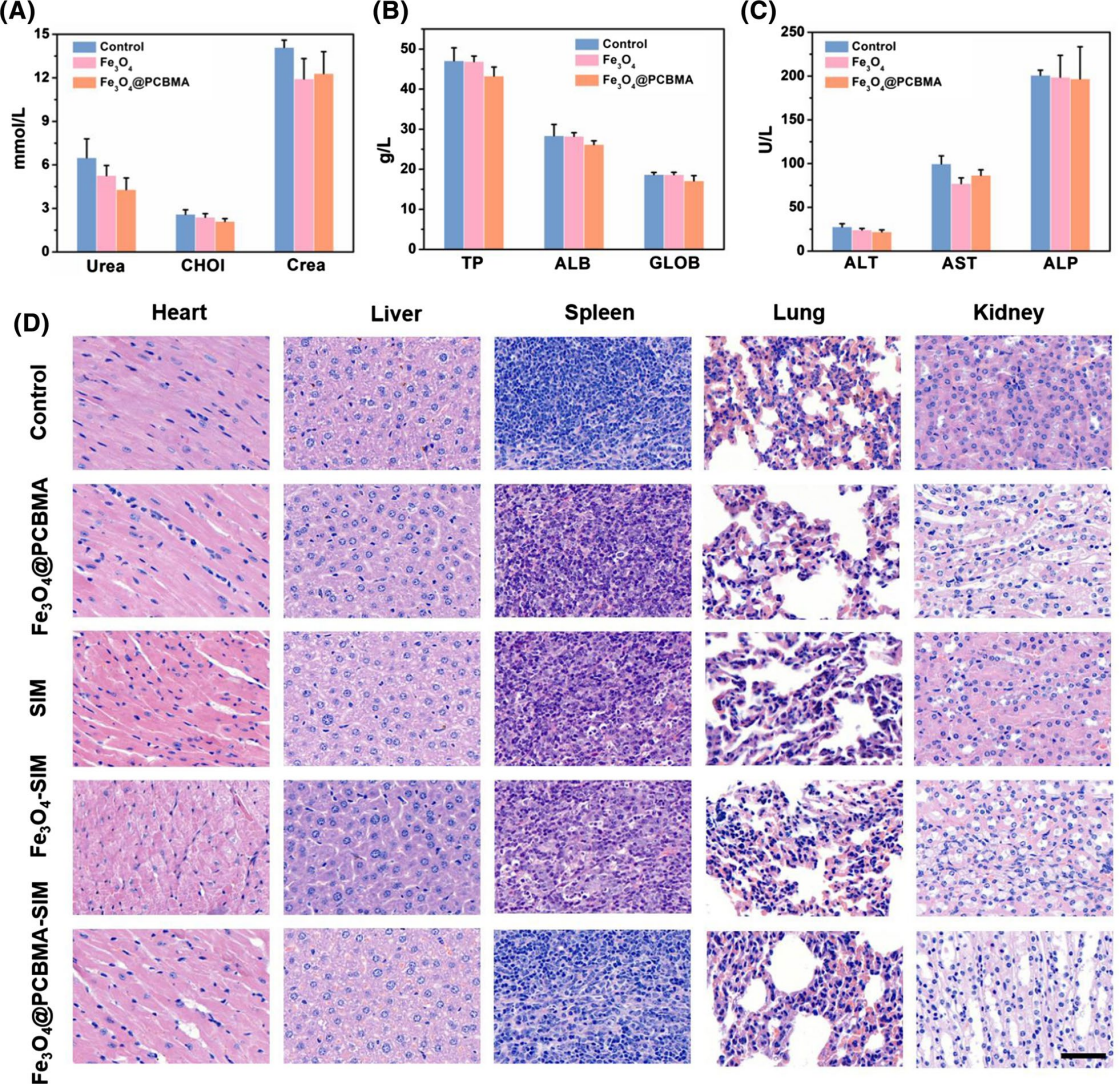
Blood biochemistry indices of renal and hepatic function including alkaline phosphatase (ALP), alanine aminotransferase (ALT), aspartate aminotransferase (AST) (**A**); globulin (GLOB), total protein (TP), albumin (ALB) (**B**) and creatinine (CREA), cholesterol (CHOL), UREA (**C**); H&E-stained slices of liver spleen, heart, lung, and kidney from each group (**D**), Scale bars were 50 μm

Encouraged by the excellent tumor accumulation of Fe_3_O_4_@PCBMA, the in vivo antitumor efficacy was then performed on MDA-MB-231 tumor models. All mice were divided into five groups (n = 5). As shown in Fig. 5C, there is no obvious change in tumor size between Fe_3_O_4_@PCBMA group and control group, while tumors grew slower in SIM and Fe_3_O_4_-SIM than control group. When encapsulated with PCBMA, the growth of tumors suppressed in 22 days and the antitumor rate was 76.1%, which indicated that TNBC could be suppressed under ferroptosis. The most impressive thing is that no significant weight loss was seen in all groups of mice (Fig. 5D), indicating that the safety of Fe_3_O_4_@PCBMA-SIM. The tumor photos in all groups were consistent with the tumor growth curves. To further study the systemic toxicity of the nanoparticles, all mice were sacrificed at the 22th day treatment and the main organs (liver, spleen, heart, lung, and kidney) were removed and co-stained by hematoxylin and eosin (H&E). As shown in Fig. 6D, there were no obvious tissue damages and noticeable pathological changes in the five groups, which indicated that nanoparticles were possess biosafety and could be an effective nanoplatform for cancer treatment. In addition, TUNEL assay results showed that Fe_3_O_4_@PCBMA-SIM group had the highest mortality in vivo tumor treatment and it is consistent with its good antitumor inhibition effect (Additional file 1: Fig. S9). In addition, blood biochemistry and routine blood tests were used to tasty the of Fe_3_O_4_@PCBMA. As shown in Fig. 6A–C, there was no obvious difference of blood indices compared Fe_3_O_4_ and Fe_3_O_4_@PCBMA group with the control group. Moreover, there is no difference between nanoparticles treatment group and control group of the whole blood panel analysis result (Additional file 1: Fig. S10). Therefore, Fe_3_O_4_@PCBMA had good biocompatibility without liver and kidney toxicity.

## Conclusion

Ferroptosis is a key tumor suppression mechanism. Herein, we presented the ferroptosis nanomedicine by loading simvastatin (SIM), a ferroptosis drugs, into zwitterionic polymer coated of magnetic nanoparticles (Fe_3_O_4_@PCBMA), thereby improving the therapeutic effect of triple negative breast cancer. This drug delivery platform was demonstrated to have higher toxicity against MDA-MB-231 than MCF-7, which demonstrated that statins could effectively kill triple negative breast cancer. Furthermore, the western blot result illustrated SIM could through inhibit HMGCR to inhibit the mevalonate pathway and deactivate GPX4. With the inherited blood circulation property and ferroptosis effect, Fe_3_O_4_@PCBMA-SIM was intravenously injected into MDA-MB-231 tumor-bearing mice and their treatment efficiency in vivo was evaluated. Our finding highlights that Fe_3_O_4_@PCBMA-SIM exhibit an excellent tumor suppression, which will open an avenue of triple negative breast cancer therapy.

## Materials and methods

### Synthesis of magnetic nanoparticles and modification

Fe_3_O_4_ nanoparticles were prepared by the solvothermal reaction method [38]. Typically, FeCl_3_·6H_2_O (0.54 g, 2 mM), sodium acetate (1.2 g, 15 mM), sodium citrate dihydrate (0.24 g, 1 mM) and 20 mL ethylene glycol mixed and stirred for 30 min. Then transferring the above solution into a three-necked flask (100 mL) under 200 ℃ for 10 h. Finally, the resulting products were dispersed in 50 mL of deionized water for further use.

Before encapsulating the zwitterionic polymer, 200 mg of Fe_3_O_4_, 5 mL of deionized water, 1.5 mL of aqueous ammonia solution (28–30%) was mixed with 35 mL of ethanol and stirred at 60 °C for 30 min. Then, 300 mg of MPS was slowly added for another 12 h. Finally, the obtained products were washed three times with ethanol and were named as Fe_3_O_4_-MPS.

### Synthesis of carboxybetaine methacrylate (CBMA) monomer

In a typical recipe [31], 3.14 g of 2-(dimethylamino) ethyl methacrylate was dissolved in 25 mL of anhydrous acetone, to which 1.5 mL of β-propiolactone was added and stirred at 4 °C for 6 h. The CBMA monomer was obtained by extraction filtration and washed with diethyl ether.

### Synthesis of Fe_3_O_4_@PCBMA

Fe_3_O_4_@PCBMA were prepared by modified reflux-precipitation polymerization method [39]. Using a typical method, 5 mL ethanol, 4 mg of AIBN, 20 mg of Fe_3_O_4_-MPS, 5 mg of BAC, 95 mg of CBMA monomer and 35 mL of acetonitrile were added into a three-necked flask and stirred at 90 °C for 1 h under N_2_. Then the resulting product washed with ethanol and deionized water and obtained the composite nanoparticles, denoted as Fe_3_O_4_@PCBMA.

### SIM loading of Fe_3_O_4_@PCBMA (Fe_3_O_4_@PCBMA-SIM)

Firstly, 3 mg of SIM, 1 mL of deionized water, 2 mL ethanol and 10 mg of Fe_3_O_4_@PCBMA were mixed. The above solution was stirred in the dark for 24 h at room temperature and then vacuum-rotary evaporation to remove solvent and washed with water. Finally, UV–vis spectrophotometer was used to calculate the loading rate of SIM at 238 nm.

### SIM releasing from Fe_3_O_4_@PCBMA-SIM

10 mg of Fe_3_O_4_@PCBMA-SIM were dispersed into 5 mL of GSH with different concentrations (0, 5, 10 mM). Afterwards, the mixed solution was stirred at 37 °C under dark conditions. After a specific time interval, centrifuge for collecting the supernatant and added another 5 mL of GSH solution. The SIM releasing amount was calculated according to the UV–vis absorbance value of SIM in the supernatant.

### The degradation of Fe_3_O_4_ and Fe_3_O_4_@PCBMA

To measure the degradation of nanoparticles, Fe_3_O_4_ and Fe_3_O_4_@PCBMA (100 µg/mL) were placed in a 1.4 × 10^4^ Dalton dialysis bag. Afterwards, the dialysis bag was dip in 200 mL of PBS (pH 5.0, 6.5, 7.4) with different concentrations of GSH (0 mM, 10 mM). And then, 2 mL of solution was removed and added 2 mL of PBS with GSH at a certain point in time. ICP-AES was used to measure the amount of Fe.

### In vitro cytotoxicity assay

The cytotoxicity of Fe_3_O_4_, Fe_3_O_4_@PCBMA, free SIM, Fe_3_O_4_-SIM and Fe_3_O_4_@PCBMA-SIM were measured according to previous report [40]. Firstly, MCF-7 and MDA-MB-231 cells were incubated into 96-well plate. After 12 h incubation, different concentrations of Fe_3_O_4_, Fe_3_O_4_@PCBMA, free SIM, Fe_3_O_4_-SIM and Fe_3_O_4_@PCBMA-SIM were added respectively. Afterwards, CCK-8 was added after 24 h of culture to measuring the cell viability.

### Cell uptake assay

Before cell uptake assay, MCF-7 cells and MDA-MB-231 cells were firstly cultured in a 35-mm glass-bottomed dish. Then SIM, Fe_3_O_4_@PCBMA, Fe_3_O_4_-SIM, Fe_3_O_4_@PCBMA-SIM were added to MCF-7 cells and MDA-MB-231 cells (500 μL, 50 μg/mL). Nanoparticle untreated cells were used as control group. After culturing 4 h at 37 °C, FerroOrange probe (1 μmol/L) was added to each dish for 30 min at 37 °C and washed with PBS. Finally, CLSM was used to capture the fluorescence images. (CLSM, Ex: 561 nm, Em: 570–620 nm).

### Measuring the reactive oxygen species (ROS) and lipid hydroperoxides (LPO) generation

To investigate the ROS generation, MCF-7 cells and MDA-MB-231 cells were cultured in a 35-mm glass-bottomed dishes. After 24 h incubation, SIM, Fe_3_O_4_@PCBMA, Fe_3_O_4_-SIM, Fe_3_O_4_@PCBMA-SIM suspension (500 μL, 50 μg/mL) were added to each dish and continue cultivation. Then, DCFH-DA (an ROS probe, Ex: 488 nm, Em: 537 nm) was added to each dish after 6 h cultivation for measuring reactive oxygen species (ROS) produced by cells. CLSM was used to capture the fluorescence images of cells.

In addition, MCF-7 cells and MDA-MB-231 cells were also seeded in a 6-well plate for the flow cytometry measurement. Similarly, nanoparticles (500 μL, 50 μg/mL) were added to 6-well plate. DCFH-DA (ROS probe) and C11-BODIPY (LPO probe) were used to stain above cells. Finally, all cells were digested for flow cytometry measurement.

### In vivo biodistribution and pharmacokinetics of Fe_3_O_4_@PCBMA

Before biodistribution and pharmacokinetics of Fe_3_O_4_@PCBMA nanoparticles, a subcutaneous tumor model of MDA-MB-231 cells was firstly constructed. After the tumor volume reached 60 mm^3^, the tumor-bearing nude mice were treated with Fe_3_O_4_ and Fe_3_O_4_@PCBMA (2 mg/mL) through injected intravenously. After 12 h, 24 h and 48 h, spleen, Heart, kidney, liver, lung and tumor were taken out and dissolved by acid mixture (Vperchloric acid:Vhydrochloric acid = 1:4). After diluted with deionized water and filtered through a 0.22 mm membrane, iron distribution in tissues were measured by ICP-AES.

Similarly, eight Balb/c nude mice (male, 20–22 g) were used to measure the pharmacokinetics of Fe_3_O_4_@PCBMA. Firstly, Fe_3_O_4_ and Fe_3_O_4_@PCBMA (2 mg/mL) were intravenously injected into female mice (n = 3). Then, the whole blood (30 μL) was extracted through orbital sinus at different time points (0, 15 min, and 1, 2, 4, 8, 12, 24, 48 h) and dissolved by acid mixture as above for detected iron distribution in blood.

### In vivo therapy of ferroptosis

To test the therapy of ferroptosis in vivo, MDA-MB-231 cells were injected to subcutaneous of mice to build a tumor model. When the tumor volume reaches about 60 mm^3^, all mice were randomly divided into five groups (n = 5) for various treatments. Then, mice were treated with PBS, Fe_3_O_4_@PCBMA, SIM, Fe_3_O_4_-SIM and Fe_3_O_4_@PCBMA-SIM through injected intravenously. The injected doses of SIM were 4 mg/kg body weight in each mouse on days 0, 3, 6, and 9. Date was collected every other day for recording the change of body weight and tumor volume after drugs treated. After 22 days of therapy, the organs (liver, kidneys, spleen, lung, heart and tumor) of each group mice were taken out and dispersed in 4% paraformaldehyde for H&E stain. Finally, TUNEL assay was used to measurement the apoptotic cells in the tumor slices.

### Blood biochemistry and routine blood testing

To measure the safety of nanomedicine, 12 female ICR mice (25–28 g) were injected intravenously with 100 μL of PBS, Fe_3_O_4_ and Fe_3_O_4_@PCBMA. After 24 h, blood sample from each mouse were taken out and measured the biochemical and routine blood testing indexes to test the safety of nanoparticles.

### Statistical analysis

The data were showed as mean ± standard deviation. The differences between groups were performed by one-way ANOVA with Dunnett’s multiple comparisons test. P < 0.05 was considered statistically significant.

## Supplementary Information

**Additional file 1: Fig. S1**. FT-IR spectra of Fe_3_O_4_ and Fe_3_O_4_-MPS nanoparticles. **Fig. S2.** TEM images of Fe_3_O_4_@PCBMA-SIM nanoparticles (A); SEM images of Fe_3_O_4_@PCBMA-SIM nanoparticles (B). **Fig. S3.** DLS diameters of Fe_3_O_4_@PCBMA-SIM nanoparticles dispersed in water, PBS, bovine serum albumin (BSA) and Dulbecco’s Modified Eagle Medium (DMEM). **Fig. S4.** The degradation property of Fe_3_O_4_ nanoparticles dispersed in different concentrations of GSH (0 mM and 10 mM) and pH values (5.0, 6.5 and 7.4). **Fig. S5.** Cell viability of ferrous sulfate to MDA-MB-231 cells. **Fig. S6.** Cell viability of ferrous sulfate to MCF-7 cells. **Fig. S7.** Biodistribution of Fe_3_O_4_ and Fe_3_O_4_ @PCBMA nanoparticles after 12 h injection. **Fig. S8.** Biodistribution of Fe_3_O_4_ and Fe_3_O_4_ @PCBMA nanoparticles after 48 h injection. **Fig. S9.** TUNEL staining of tumor tissues. Scale bars was 20 μm. **Fig. S10.** Whole blood panel analysis of nanoparticle-treated mice at 24 h post-injection.

## Data Availability

All data generated or analyzed during this study are included in this published article.
