# Supplementary material for: Simvastatin induced ferroptosis for triple-negative breast cancer therapy
Source: J Nanobiotechnology. 2021 Oct 9;19:311. doi: 10.1186/s12951-021-01058-1 (PMC8502296; doi:10.1186/s12951-021-01058-1)
Supplement: Supplementary file 1 — Additional file 1: Fig. S1. FT-IR spectra of Fe3O4 and Fe3O4-MPS nanoparticles. Fig. S2. TEM images of Fe3O4@PCBMA-SIM nanoparticles (A); SEM images of Fe3O4@PCBMA-SIM nanoparticles (B). Fig. S3. DLS diameters of Fe3O4@PCBMA-SIM nanoparticles dispersed in water, PBS, bovine serum albumin (BSA) and Dulbecco’s Modified Eagle Medium (DMEM). Fig. S4. The degradation property of Fe3O4 nanoparticles dispersed in different concentrations of GSH (0 mM and 10 mM) and pH values (5.0, 6.5 and 7.4). Fig. S5. Cell viability of ferrous sulfate to MDA-MB-231 cells. Fig. S6. Cell viability of ferrous sulfate to MCF-7 cells. Fig. S7. Biodistribution of Fe3O4 and Fe3O4 @PCBMA nanoparticles after 12 h injection. Fig. S8. Biodistribution of Fe3O4 and Fe3O4 @PCBMA nanoparticles after 48 h injection. Fig. S9. TUNEL staining of tumor tissues. Scale bars was 20 μm. Fig. S10. Whole blood panel analysis of nanoparticle-treated mice at 24 h post-injection. [file 12951_2021_1058_MOESM1_ESM.docx]

Simvastatin induced ferroptosis for human triple-negative breast cancer therapy

Xianxian Yao^1^, Ruihong Xie^1^, Yongbin Cao^1^, Jing Tang^2^, Yongzhi Men^3^, Haibao Peng ^4^* and Wuli Yang^1^*

^1^ State Key Laboratory of Molecular Engineering of Polymers & Department of Macromolecular Science, Fudan University, Shanghai, 200433, China

^2^ Department of Materials Science and Engineering, Stanford University, Stanford, CA, United States

^3^ Shanghai General Hospital, Shanghai Jiao Tong University School of Medicine. Shanghai, 200080, China

^4^ Institute for Translational Brain Research, Fudan University, Shanghai 200032, China

^*^Corresponding authors: E-mail address: [haibaopeng@gmail.com](mailto:haibaopeng@gmail.com) (Hai-Bao Peng); [wlyang@fudan.edu.cn](mailto:wlyang@fudan.edu.cn) (Wu-Li Yang)

**Materials**

2-azobisisobutyronitrile (AIBN, 99%), 2-(Dimethylamino) ethyl methacrylate， diethyl ether，β-propiolactone and 3-(Trimethoxylsilyl) propyl methacrylate (MPS, 99%) were purchased from Sigma-Aldrich. 3-aminopropyltriethoxysilane (MPS) was purchased from Alfa Aesar. Acetonitrile and anhydrous acetone were purchased from Sinopharm Chemical Reagent Co. Ltd. Sodium citrate dehydrate, ferric chloride hexahydrate (FeCl_3_·6H_2_O), sodium acetate, ethylene glycol and N, N′-bis-(acryloyl) cystamine (BAC) was purchased from Alfa Aesar (UK). Dulbecco's Modified Eagle Medium (DMEM/High Glucose 1X) was purchased from GE Healthcare Life Science (Hyclone™, Pittsburgh, USA). Fetal bovine serum (FBS) and trypsin-ethylene diamine tetraacetic acid (TrypsinEDTA, 0.05%) were purchased from Life Science (Gibco™, Pittsburgh, USA). DCFH-DA reactive oxygen species assay kit and the CCK-8 were bought from Nanjing Key Gen Biotech Inc. FerroOrange probe was obtained from Dojindo Laboratories, Kumamoto, Japan. C11-BODIPY was purchased from Thermo Fisher Scientific. Ultrapure water was obtained from a Millipore pure water system. All chemicals were of analytical grade and were used without further purification.

**Instrumentation**

Dynamic light scattering (DLS) and zeta potential were measured at 25 °C on a Zetasizer Nano ZS90 analyzer (Malvern Instruments, Ltd., UK). The Fourier transform infrared (FT-IR) spectra were obtained using a Thermo-Fisher Nicolet 6700 Fourier transform infrared spectrometer with KBr pellets. UV-vis spectra were recorded at 25 Con PerkinElmer Lamba 750 spectrophotometer. Scanning electron microscopy (SEM) images were recorded on a scanning electron microscopy (Ultra 55, Zeiss, Germany) operates at 5 kV. Transmission electron microscope (TEM) images were taken on a transmission electron microscope (Tecnai G2 20 TWIN, FEI, USA) operated at an acceleration of 200 kV by dropping the dispersion onto a carbon-coated copper grid. Confocal laser scanning microscopy (CLSM) images were obtained using a Nikon C2+ laser scanning confocal microscope (Nikon, Japan). Flow cytometry analysis was performed at 37 °C on a Beckman Coulter Gallios flow cytometer. Inductively coupled plasma spectrometry (ICP-AES) was carried out on a Thermo Scientific iCAP 7400 series ICP-AES instrument.

**Cell culture and mice breed**

Human breast cancer cell line (MCF-7 cells) and human triple-negative breast cancer (MDA-MB-231 cells) were purchased from Shanghai Meixuan Biotechnology Co., Ltd. (China) and cultured in Dulbecco Minimum Essential Medium (DMEM) supplemented with 10% (v/v) FBS and 1% antibiotics (penicillin/streptomycin, 100 U/ mL). Cells were incubated in a 5% CO_2_ atmosphere at 37 °C.

Female Balb/c nude and ICR (Institute of Cancer Research) mice (20-22 g) were purchased from Shanghai BK Lab. Animal Research Center and raised under standard conditions at 25 ± 2 °C under 60% ± 10% humidity and a 12-h light/12-h dark cycle. All procedures for animal experiments were carried out under the guidelines approved and supervised by the ethics committee of Fudan University.

**Glutathion peroxidase 4 (GPX4) protein and 3-hydroxy-3-methylglutaryl coenzyme a reductase (HMGCR) protein analysis by western blot**

To investigate the GPX4 protein and HMGCR protein expression, MCF-7 cells and MDA-MB-231 cells were seeded into 6-well plate, cultured for 12 h, and added to nanoparticles (500 μL, 100 μg/mL). After culturing for 12 h, the nanoparticles were washed three times with PBS and cells were lysed on ice in the lysis buffer, containing 1% protease inhibitors and phosphatase inhibitors (Sangon Biotech, Shanghai). After centrifugation at 12000 rpm for 10 min, supernatants were collected. Protein concentrations in supernatants were determined by BCA method (Bio-Rad, Hercules, CA, USA). Samples were separated on 10% sodium dodecyl sulfate polyacrylamide gel electrophoresis, and transferred to polyvinylidene difluoride (PVDF) membranes (Millipore, Billerica, MA, USA). After blocking with skimmed milk in TBST (TBS with 0.1% Tween-20, pH 7.6) for 1 h, membranes were incubated with rabbit anti-CBS antibody (1:1000, UK) at 4 °C overnight. After washing three times with TBST, the membranes were further incubated with horseradish peroxidase conjugated secondary antibodies for 1 h. Protein brands of interest were detected by chemiluminescence substrate (Millipore) using Amersham Image 600 (GE, USA).

**Methods**

The percentage of injected nanoparticle dose per gram of tissue (% ID/g)

The percentage of injected nanoparticle dose per gram of tissue (% ID/g) was calculated using the following formula [S1]:

$$\% ID/g= \frac{dose in tissues}{injected dose \times weight of tissue (g)} \times100$$

Where the nanoparticle dose in tissues was determined by measuring the concentration of iron ion of Fe_3_O_4_@PCBMA nanoparticles using an inductively coupled plasma spectrometer (ICP).





**Fig. S1.** FT-IR spectra of Fe_3_O_4_ and Fe_3_O_4_-MPS nanoparticles.


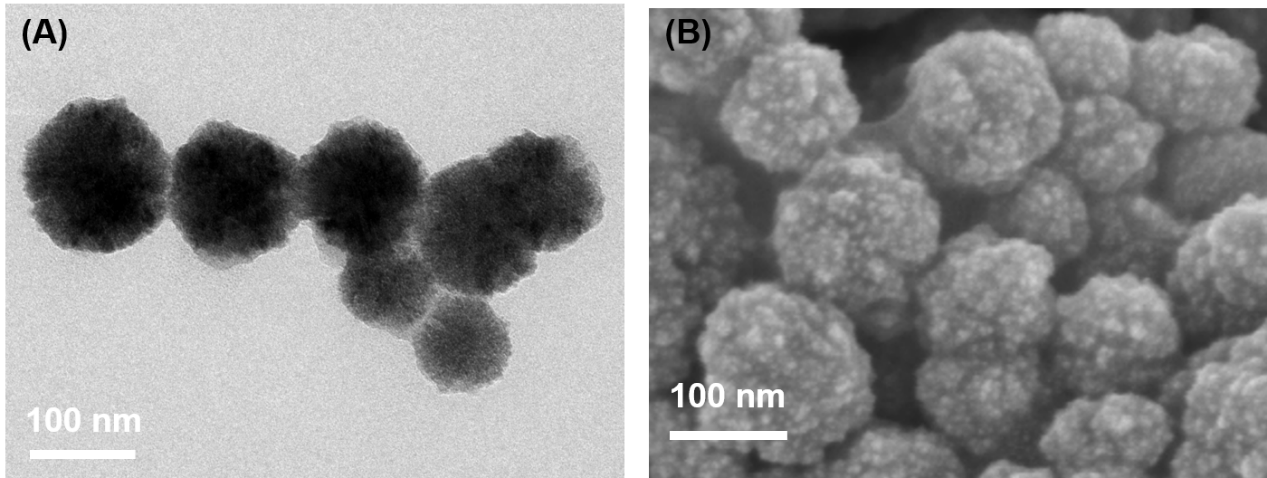


**Fig. S2.** TEM images of Fe_3_O_4_@PCBMA-SIM nanoparticles (A); SEM images of Fe_3_O_4_@PCBMA-SIM nanoparticles (B).


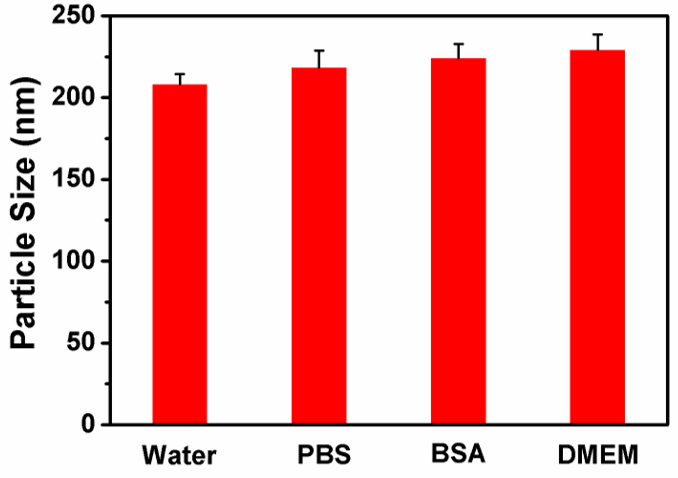


**Fig. S3.** DLS diameters of Fe_3_O_4_@PCBMA-SIM nanoparticles dispersed in water, PBS, bovine serum albumin (BSA) and Dulbecco’s Modified Eagle Medium (DMEM).





**Fig. S4.** The degradation property of Fe_3_O_4_ nanoparticles dispersed in different concentrations of GSH (0 mM and 10 mM) and pH values (5.0, 6.5 and 7.4).


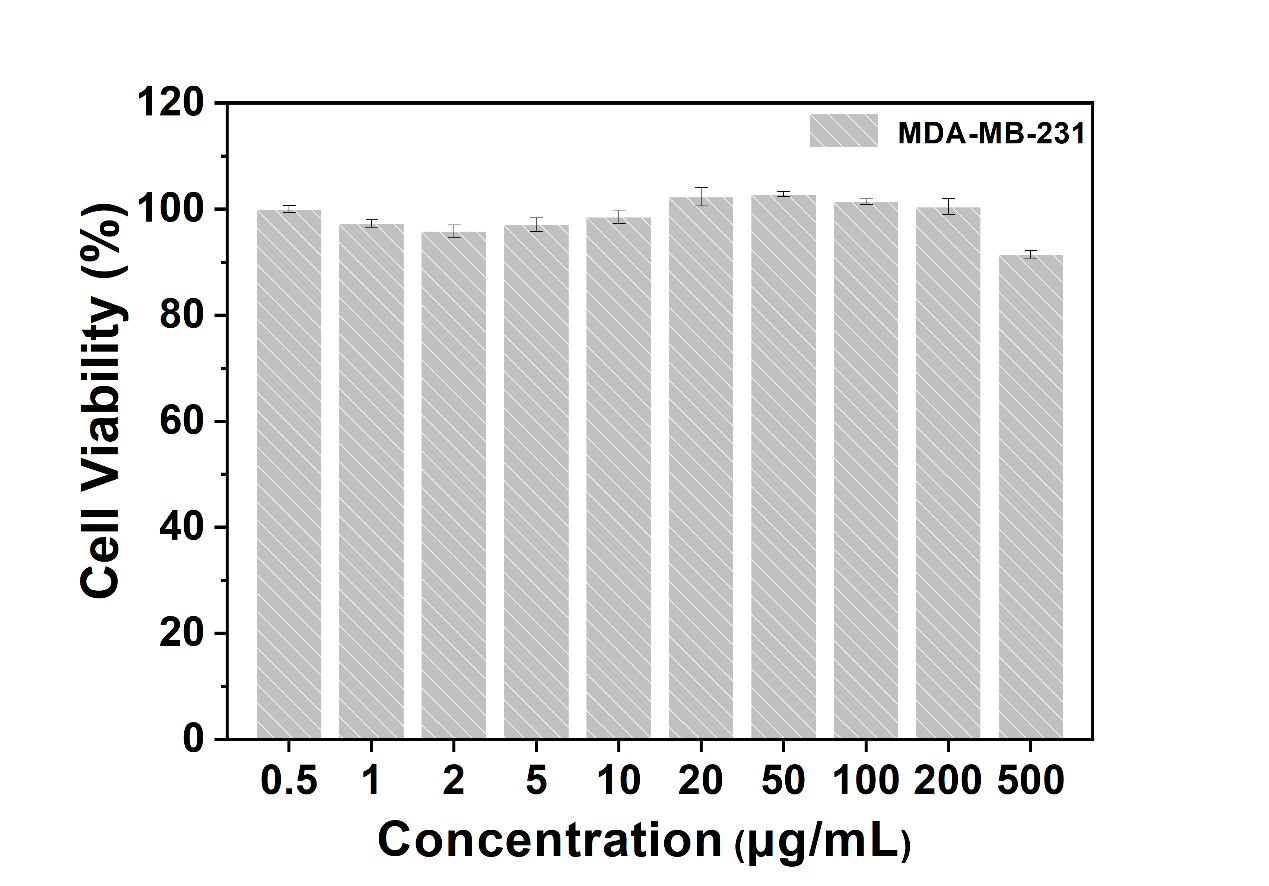


**Fig. S5.** Cell viability of ferrous sulfate to MDA-MB-231 cells.


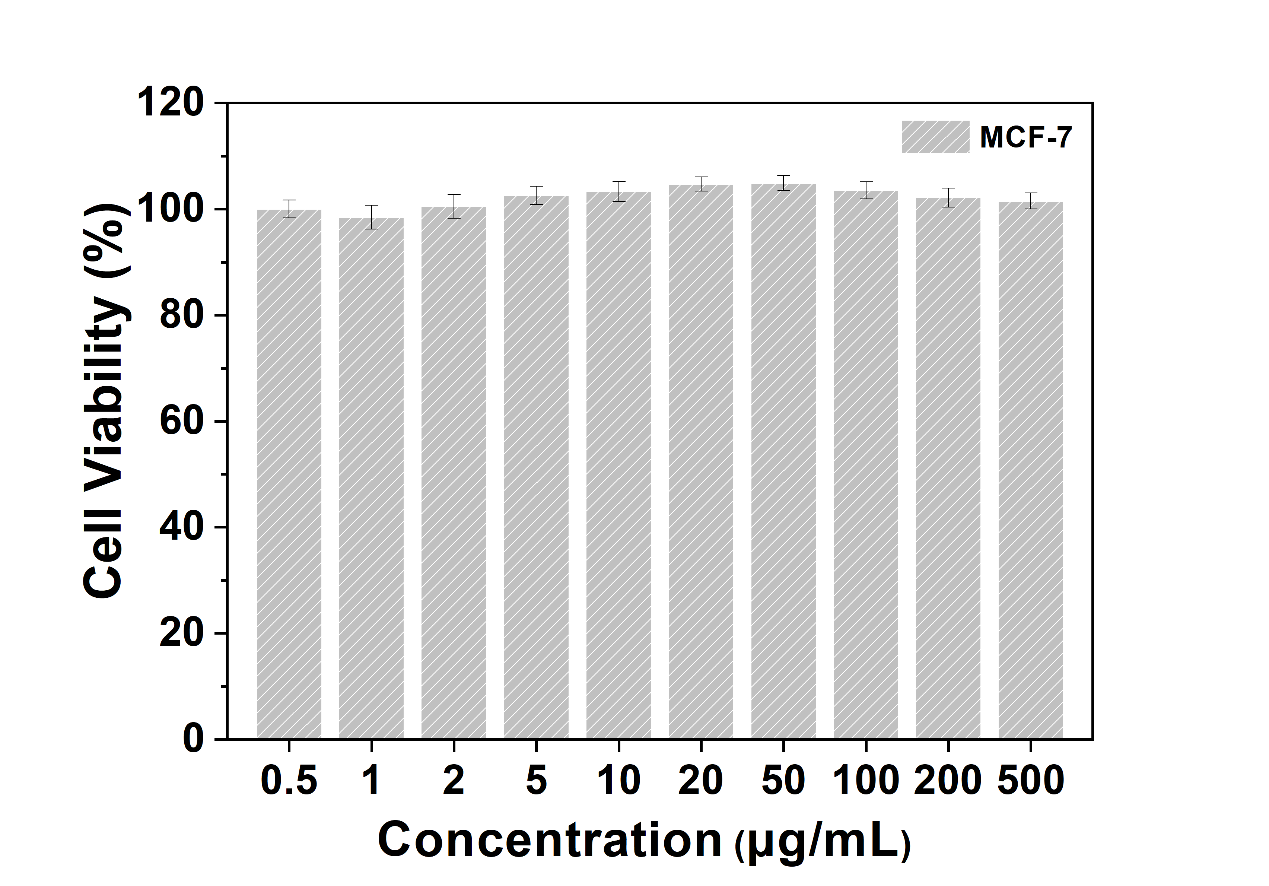


**Fig. S6.** Cell viability of ferrous sulfate to MCF-7 cells.





**Fig. S7.** Biodistribution of Fe_3_O_4_ and Fe_3_O_4_@PCBMA nanoparticles after 12 h injection; *In vivo* blood retention profiles of Fe_3_O_4_ and Fe_3_O_4_@PCBMA nanoparticles (1 mg/mL, 100 μL).

**

**

**Fig. S8.** Biodistribution of Fe_3_O_4_ and Fe_3_O_4_@PCBMA nanoparticles after 48 h injection; *In vivo* blood retention profiles of Fe_3_O_4_ and Fe_3_O_4_@PCBMA nanoparticles (1 mg/mL, 100 μL).


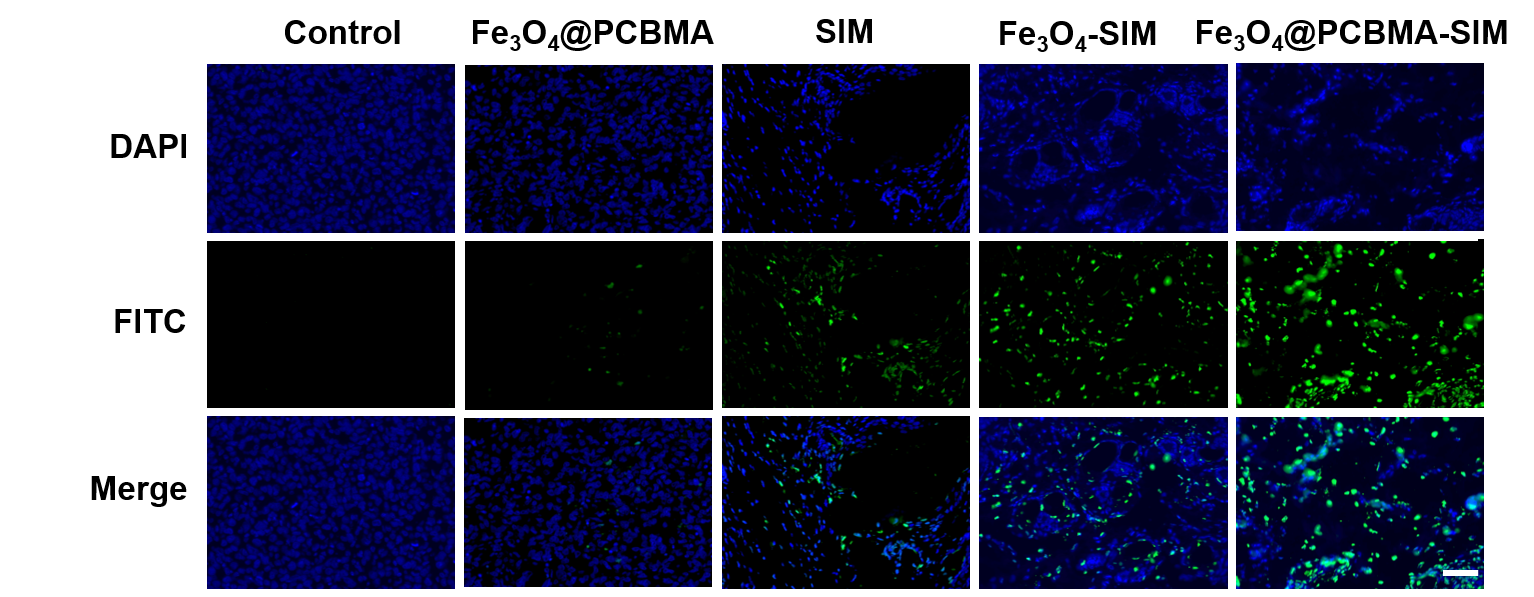


**Fig. S9.** TUNEL staining of tumor tissues. Blue and green fluorescence were living cells and apoptotic cells, respectively. Scale bars was 20 μm.

**
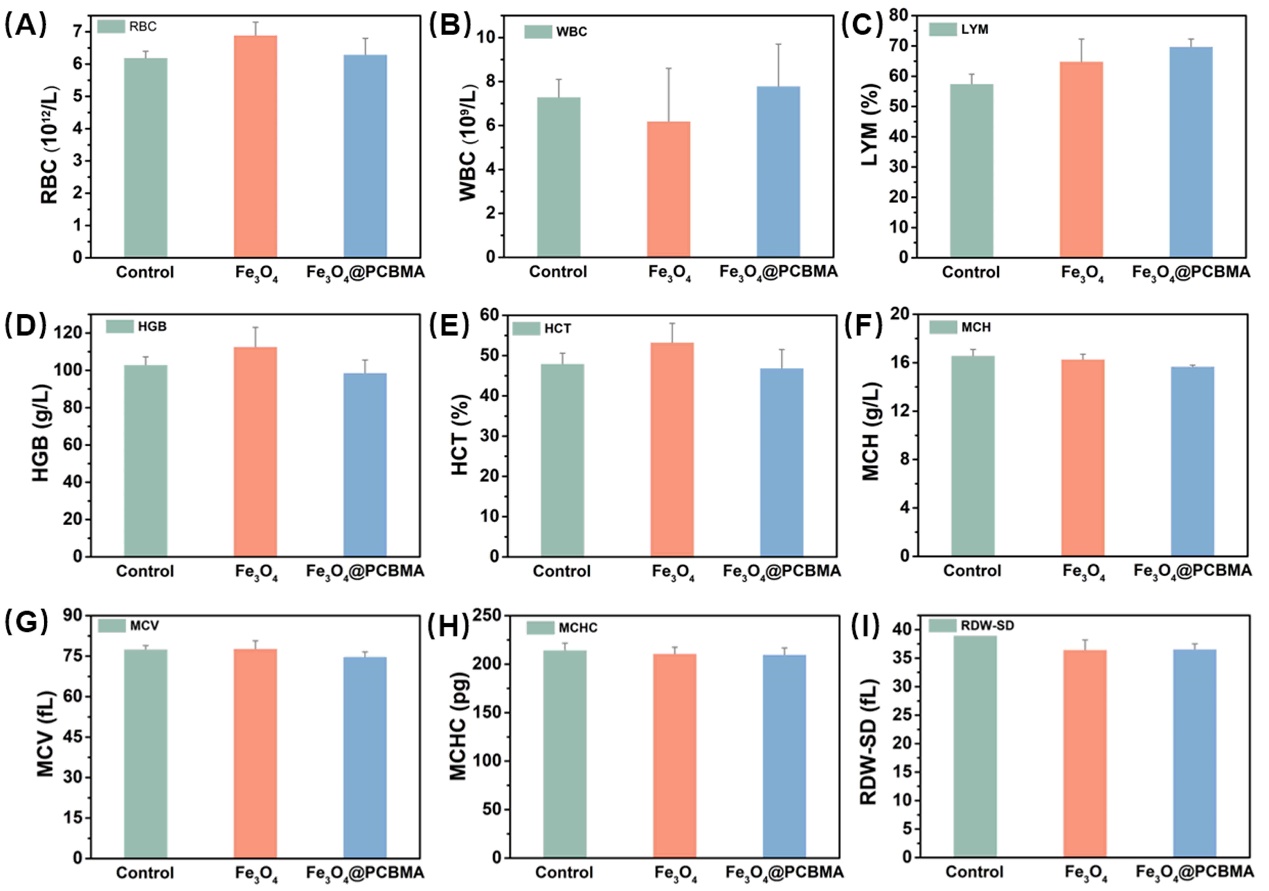
**

**Fig. S10.** Whole blood panel analysis of nanoparticle-treated mice at 24 h post-injection. Normal mice without treatment were used as negative controls. Data are the means ± SD. n=4. WBC, white blood cell; RBC, red blood cell; HGB, hemoglobin; HCT, hematocrit; MCV, mean corpuscular volume; MCH, mean corpuscular hemoglobin; MCHC, mean corpuscular hemoglobin concentration; RDW, red blood distribution width; PLT, platelets.

1. Guo X, Shi CL, Yang G, Wang J, Cai ZH, Zhou SB. Dual-responsive polymer micelles for target-cell-specific anticancer drug delivery. Chem. Mater. 2014;26: 4405-4418.
